# Influence of Tributary Inflows on Sediment Bacterial Community Composition of the River Mainstem

**DOI:** 10.3390/microorganisms14050984

**Published:** 2026-04-28

**Authors:** Ioana Boeraș, Ana Maria Benedek, Angela Curtean-Bănăduc, Doru Bănăduc

**Affiliations:** Biology and Ecology Research Center, Faculty of Sciences, Lucian Blaga University of Sibiu, 550012 Sibiu, Romania; ana.benedek@ulbsibiu.ro (A.M.B.); ad.banaduc@yahoo.com (D.B.)

**Keywords:** microbiome, lotic, sediment, main river, tributary

## Abstract

River sediment microbial communities are an integral part of fluvial ecosystems, where they play a central role in nutrient cycling. Although these communities share a core group of microorganisms, their overall composition can be influenced by natural environmental conditions and anthropogenic factors. While anthropogenic influences on river microbial communities have been extensively studied, natural drivers have received comparatively less attention. In this study, we evaluated the impact of tributary inflow on the microbial assemblages of a main river stem. Sediment samples were collected from both the main channel and some of its tributaries, and bacterial community composition was characterized using 16S rRNA gene amplicon sequencing. Taxonomic profiling revealed a largely shared core community typical of riverine sediments across all sites. While alpha diversity did not differ significantly between main river and tributary samples, beta diversity analyses demonstrated clear segregation between the two environments, indicating distinct community structures. Correlation analyses further showed that microbial assemblages in the main river downstream of tributary confluences were significantly associated with tributary community composition, highlighting the influence of tributary inflow on bacterial communities in the main river.

## 1. Introduction

Rivers and their associated basins are intricate, dynamic, and interconnected complexes of socio-ecological systems that strongly influence adjacent environments and human activities [1,2,3]. On the other hand, rivers and their basins are influenced by a large number of natural and anthropogenic stressors in a synergistic way [4,5]. Within lotic systems, microbial communities are foundational to ecosystem function, acting as the primary drivers of biogeochemical cycles [6,7,8,9]. They play critical roles in nutrient transformation, energy flow, trophic transfer, and water self-purification [10]. Although microorganisms are major drivers of river ecosystem functioning, their community composition and structure are themselves shaped by environmental conditions [11,12]. To date, most studies have emphasized the effects of anthropogenic pressures on riverine microbial communities, whereas comparatively fewer have examined their natural or intrinsic organization [13]. A comprehensive understanding of external impacts on microbial communities therefore requires prior characterization of intrinsic drivers, such as river flow dynamics and the influence of tributaries. While sediment microbial communities have been extensively studied—largely in the context of anthropogenic disturbance—the relationships between microbial communities in main river channels and their tributaries remain insufficiently explored.

Previous studies have primarily focused on the spatial distribution of microbial communities within river main stems [14,15,16,17], whereas changes in microbial composition and diversity across stream orders—including headwater streams, tributaries, and main stems—remain poorly characterized. Liu et al. show that the structure of bacterial communities significantly changed and their beta diversity increased along the streams, tributaries, and main stems of rivers [13]. Moreover, tributary inflows can substantially influence the microbiome of the main river by introducing distinct bacterial assemblages, as well as additional nutrients and organic matter [18]. Consequently, tributaries may act as key drivers shaping mainstem microbial community structure, diversity, and metabolic function.

The Mureș River represents a major hydrological and ecological corridor in Eastern Europe, playing a critical role in regional biodiversity, water resources, and human activities. Its hydrographical network is subject to continuous and diverse anthropogenic pressures that affect its ecological dynamics, quality, and balance, highlighting the urgent need for complex assessment and monitoring research [5,19,20]. The Mureș River is the second longest river in Romania, after the Danube, with a total length of 789 km, of which 761 km are within Romania, and it drains a basin covering 30,332 km^2^ in total, including 27,890 km^2^ within Romanian territory. The river originates in the Hășmașu Mare Mountain range of the Eastern Carpathians and flows east to west across Romania before entering Hungary, where it discharges into the Tisza River. The Mureș River basin encompasses a wide range of geomorphological units, from mountainous regions through hills and plateaus, transitioning downstream into valleys, meadows, and plains [21,22]. Along its course, the Mureș River receives numerous tributaries, the most significant of which are the Arieș River on the right bank and the Târnava, Sebeș, and Strei rivers on the left bank. The Arieș River drains predominantly karstic mountain areas, whereas the Târnava River drains volcanic terrains [20].

Studies on the dynamics and characteristics of microbial communities in the Mureș River basin remain scarce [23,24], highlighting the need for continued research to address this gap in this human-impacted hotspot [25]. This study aimed to evaluate the influence of tributary-derived microbial communities on the sediment microbiome of the main channel of the Mureș River, an important river system in Eastern Europe. Sediment samples were collected from the main stem and several major tributaries, and bacterial community composition was characterized using 16S rRNA gene amplicon sequencing. Potential differences in the microbial communities from the main river versus the tributaries were assessed by looking at the communities’ structure at the phylum and class levels as well as alpha and beta diversities. We hypothesized that (i) the bacterial community structure and alpha diversity of the main river differ significantly from those of its tributaries, (ii) beta diversity of community structures is influenced by the relative position and distance between sampling sites, and (iii) tributaries act as sources of microbial taxa that are transported downstream, thereby contributing to and potentially reshaping the microbial community structure of the main river sediments. Results show that although no significant differences were detected in bacterial alpha diversity, bacterial community structure differed between the main river and tributaries, as indicated by differences in beta diversity. Furthermore, correlation analyses revealed a shift in bacterial community structure immediately downstream of tributary confluences, with downstream mainstem sites exhibiting greater similarity to the corresponding tributaries than to upstream mainstem sites. Collectively, these results highlight the influence of tributary-derived microbial communities on the bacterial assemblages of the main river.

## 2. Materials and Methods

### 2.1. Sampling and Storing

**Figure 1:**
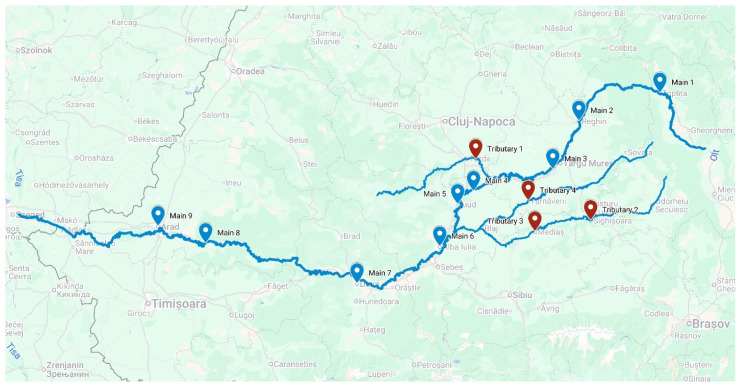
Map of the Mureș River and tributaries showing sampling locations. Sampling sites on the main river are labeled blue while sampling sites on the tributaries are labeled red.

Samples were collected from nine sites along the Mureș River and four sites on the tributaries. All samples were collected in the same season (winter of 2018–2019). Sampling locations were chosen upstream of cities or villages so as to have a more natural environment without immediate anthropogenic impact (Figure 1). Sediment samples were collected in sterile bags and stored in a portable refrigerator for transportation to the laboratory, where they were kept frozen at −50 °C. Samples from the main river have previously been used in a study to assess wastewater treatment plant effluent’s influence on river microbiome composition [24].

### 2.2. Sediment Chemical Analysis

Three main nutrients: nitrates, ammonium nitrogen, and orthophosphates were quantified in the sediment samples, as described by Boeraș et al. [23]. For all three nutrients, spectrophotometric methods were used as specified in the respective protocols: SR ISO 7890-3 for nitrates, SR ISO 7150-1 September 2001 for ammonium nitrogen, and SR EN ISO 6878 August 2008 for orthophosphates [26,27,28]. The measured concentrations were used in statistical analysis as environmental variables.

### 2.3. DNA Extraction

DNA was extracted from each sample in triplicate using 500 milligrams of sediment and the Quick-DNA Fecal/Soil Microbe Miniprep kit from Zymo Research (Irvine, CA, USA). The extraction method uses bashing beads to dislodge bacteria from the sediment particles, followed by bacteria lysis and DNA separation on a column. The quantity and quality of the extracted DNA were checked by a Specord 210 plus spectrophotometer (AnalyticJena, Jena, Germany) and 0.8% agarose gel electrophoresis. Appropriate amounts of DNA were packaged and sent to LGC Group (Berlin, Germany) for next-generation sequencing on the Illumina MiSeq platform (San Diego, CA, USA).

### 2.4. 16S Metabarcoding Analysis

The gene for the 16S ribosomal RNA was amplified using primers targeting the V3-V4 variable region [29]. A library was created by amplifying the PCR products and adding adapters with barcodes for multiplexing during sequencing. Sequencing of the 16S libraries was performed on an Illumina MiSeq platform. The libraries were demultiplexed using Illumina bcl2fastq 2.17.1.14 conversion software (Illumina, San Diego, CA, USA). Reads were discarded if they were shorter than 100 nucleotides, if they were missing the barcodes, had one-sided barcodes or the barcode pairs were conflicting. However, when the barcode distances between all libraries on the lane permitted, one or two mismatches or Ns were allowed in the barcode read. After sorting, the barcode and adapter sequences were clipped. For the next steps, the sequences were analyzed using the QIIME 2 workflow [30,31]. The DADA2 plugin, which corrects amplicon sequence data and filters phiX reads and chimeric sequences [32], was used to assess the sequence quality and create the feature table. In this step, primer sequences were removed, and the forward and reverse sequences were trimmed to a maximum length of 280 and 240 nucleotides, respectively. This step generated two curated files of the amplicon sequence variants (ASVs), one file that contains the frequency and the other that contains the sequence of the ASV. To assign phylogeny to the sequences, first, they were aligned with MAFFT [33], and then a phylogenetic tree was created using FastTree 2 [34]. In the next step, using the mean ceiling, replicate sequences at each sampling site were grouped together. This step was followed by rarefaction [35] to a maximum depth of 16,294. Using a naïve Bayes method [36], a classifier was trained with the used primer pair [29] and the Greengenes 13.8 database [37] at 99% identity. The generated classifier was then used to reference our sequences with the classify-sklearn plugin [36]. The analysis generated tables of data representing the abundance of ASV, which were further assessed by statistical analysis.

### 2.5. Statistical Analysis

The statistical analysis was performed in R version 4.3.0 [38]. Figures were generated in R using the ggplot2 package [39]. Statistical significance of the difference in relative abundance in the main river versus the tributaries was determined by Student’s *t* test where data met the normality and homoscedasticity criteria, or by the nonparametric Wilcoxon rank sum test when it did not. The alpha diversity indexes Chao, ACE, Shannon, Gini–Simpson, and Pielou were determined for each site based on the data at the species level, using functions from the vegan package [40]. Significance of the difference in alpha diversity in the bacterial communities between main sites and tributaries was tested either by the t test or by the nonparametric Wilcoxon rank sum test. Beta diversity, at the species level, was expressed as either the Bray–Curtis distance or Jaccard and calculated using functions from the ape package [41]. The measured beta diversity was used to conduct a principal coordinate analysis (PCoA) using the package vegan. To determine the impact of river type and environmental variables (nitrates, ammonium nitrogen, orthophosphates) on the community structure, we carried out a distance-based redundancy analysis (dbRDA) using functions from the package vegan. Significant differences were determined by the permutation test with 999 permutations. We employed two complementary analytical approaches—principal coordinate analysis and distance-based redundancy analysis—because each serves a distinct purpose. The principal coordinate analysis is an unconstrained ordination analysis method that is good at visualizing the data and the relationship between the samples while the distance-based redundancy analysis is a constrained method that analyses the data in relation with the environmental variables, allowing us to test the significance of the results. Therefore, we used the PCoA in particular for its visual properties and the dbRDA for the statistical significance. We used linear models to test the effect of the following environmental factors: combination of river types (main or tributary), geographical distance and number of confluences between sampling sites, direction of water flow, and concentration of nutrients (nitrates, ammonium nitrogen, orthophosphates) on the sediment microbial community beta diversity (Bray–Curtis distances between sites). Using the corrplot package [42], we calculated and tested the correlation among the bacterial community composition at the species level in the sampling sites. The correlation heat map was generated.

## 3. Results

**Figure 2:**
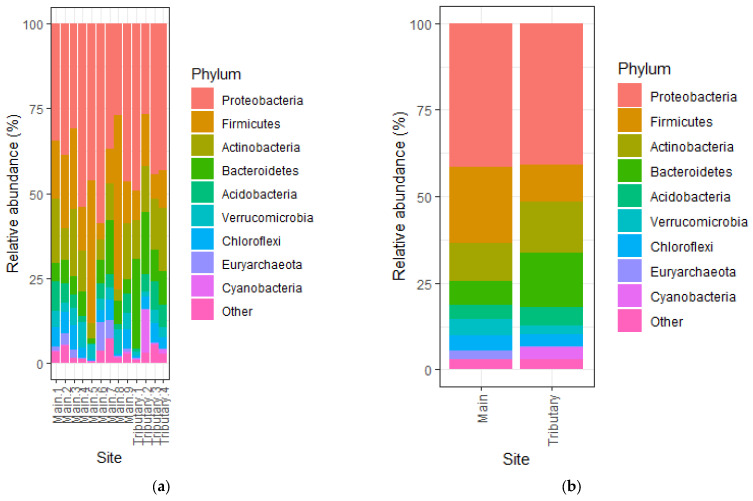
Bacterial relative abundance at the phylum level in the analyzed samples: (**a**) Abundance at each site. (**b**) Average abundance in samples from the main river versus the ones from the tributaries.

Sediment samples from the mainstream and tributaries of the Mureș River (Figure 1) were collected, and microbial DNA was extracted and analyzed by 16S amplicon sequencing. Sequencing resulted in 1,485,053 16S rRNA gene sequences after demultiplexing with an average 38,078 sequences per sample. Samples were rarefied to 16,282 sequences, and 1061 ASVs were assigned. Analysis of the microbial community structure showed some differences between sites; however, there was a common core of microbes in all samples. The most abundant phylum was Proteobacteria, representing on average 40% of all the bacteria in each sample (Figure 2a). Interestingly, the two sites with the highest abundance of Proteobacteria, over 50%, were the sites from the main river situated immediately downstream of tributaries (Figure 2a). The next best represented phylum was Firmicutes, with an average of approximately half the abundance of Proteobacteria. There were, however, two samples in the main river, Main 5 and Main 8, with a high abundance of Firmicutes, at 42% and 51% respectively (Figure 2a). When we compared the relative abundance of Phyla in the main river samples to the ones from the tributaries, we noticed significant differences in abundance for Bacteroidetes (t = −2.8309, df = 11, *p* = 0.016), Verrucomicrobia (t = 2.6153, df = 11, *p* = 0.024), Euryarchaeota (t = 2.2618, df = 8.14, *p* = 0.053), and Cyanobacteria (W = 4, *p* = 0.033) (Figure 2b). Verrucomicrobia and Euryarchaeota represented a higher percentage in the main river sites compared to the tributaries, while Bacteroidetes were better represented in the tributaries, with an average of 16%, compared to the 6% in the main river sites. Cyanobacteria was more abundant in the tributaries compared to the main sites. However, this increase was due to Tributary 2, where the relative abundance of this phylum was high, at 12% (Figure 2a).

**Figure 3:**
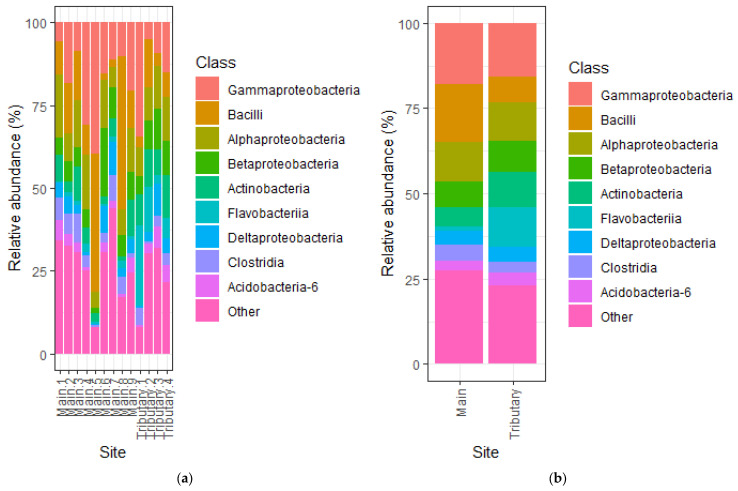
Bacterial relative abundance at the class level in the analyzed samples: (**a**) Abundance at each site. (**b**) Average abundance in samples from the main river versus the ones from the tributaries.

At the class level, there was a higher variability between abundant and less abundant classes. Due to this high variability, only Actinobacteria was significantly different (t = −2.4554, df = 11, *p* = 0.031) between main and tributary sites, with a higher abundance in the tributaries (Figure 3a). Although, on average, Bacilli was more abundant in the main river samples and Flavobacteria more abundant in the tributaries (Figure 3b), but these differences were not statistically significant (*p* > 0.05).

Alpha diversity was analyzed by computing several indices: the number of species (ASVs), ACE, Chao1, Shannon, Gini–Simpson and Pielou (Table 1). No specific trend in alpha diversity seemed to emerge. However, there were two sites from the main river that had the lowest diversity. These were the sites that had increased levels of Firmicutes—Bacilli, sites Main 5 and Main 8 (Figure 2a). When comparing alpha diversity for sites from the main river with the ones from the tributaries, we found no significant difference (*p* > 0.05).

**Figure 4:**
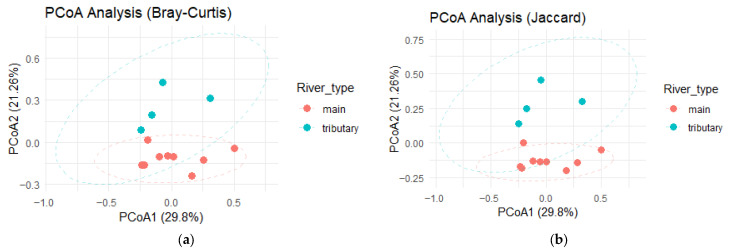
Principal coordinate analysis (PCoA) based on beta diversity indexes: (**a**) Bray–Curtis; (**b**) Jaccard.

**Figure 5:**
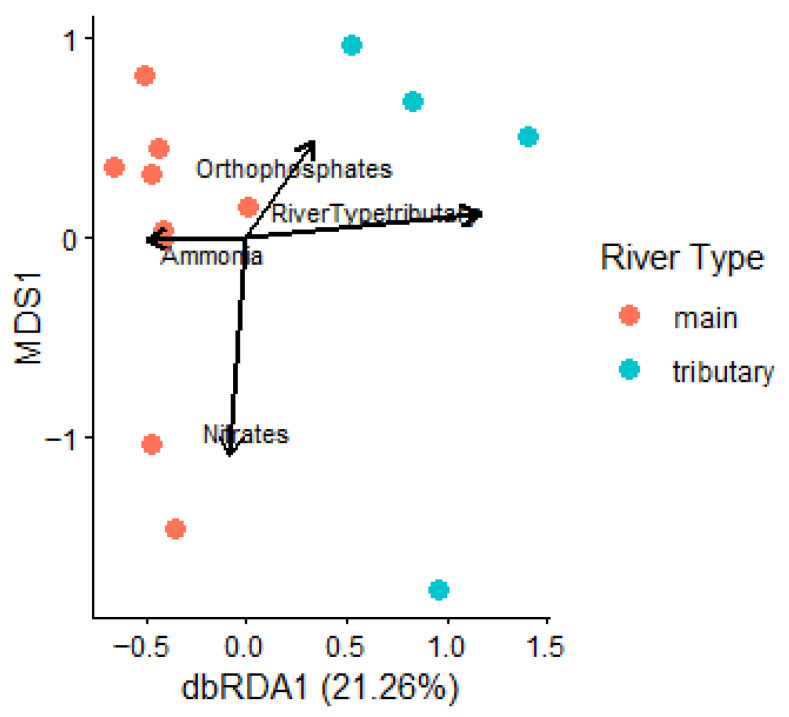
Distance-based redundancy analysis (dbRDA) ordination plot of the relationship between bacterial communities and the environmental variables.

Although the microbial communities in the main river had similar alpha diversity to the ones from the tributaries, their structure was different, as shown by the difference in beta diversity. Principal coordinate analysis (PCoA) showed separation between the main river samples and the ones from the tributaries. This analysis suggested that there is a core microbial structure in the main river that is maintained throughout the river in spite of the influences from the tributaries (Figure 4a,b). Distance-based redundancy analysis was performed to test the effect of environmental factors on the bacterial community beta diversity. Similar to PCoA, dbRDA showed significant separation between main river sites and tributaries (pseudo-F = 2.65, df = 1, *p* = 0.010) (Figure 5). In contrast, analysis of chemical composition of the sediment samples showed no significant impact on bacterial community structure. Linear regression analysis further supported the findings that the bacterial communities in the main river are different from the ones from the tributaries, as the combination of river types was the only significant predictor for beta diversity (F = 4.947, df = 2.75, *p* = 0.009), explaining 11% of its variation (R-squared: 0.116, Adjusted R-squared: 0.093).

**Figure 6:**
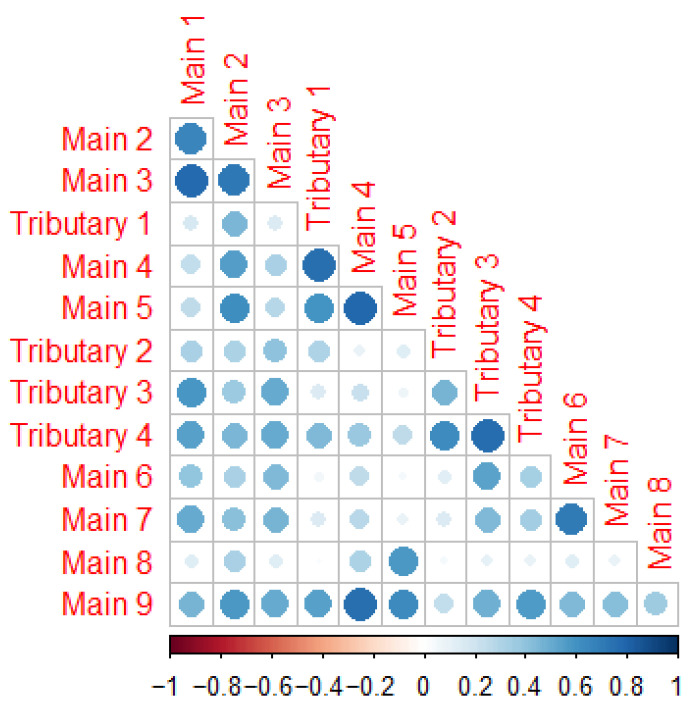
Correlation plot illustrating relationships between bacterial communities in the sampled sites.

Correlation analysis was conducted to assess the relationship between bacterial communities at the sites along the main river and the tributaries. The results indicate a significant positive correlation between microbial communities in the tributaries and the ones in the main river downstream of them. Specifically, communities in the tributaries were highly correlated with the ones from main sites downstream. However, very little correlation was observed with the main sites upstream of tributaries (Figure 6). For tributary 1, weak to no correlation was found with Main 1 (r = 0.17, *p* < 0.001), Main 2 (r = 0.45, *p* < 0.001), and Main 3 (r = 0.16, *p* < 0.001), all located upstream of the confluence. In contrast, strong correlations were observed with Main 4 (r = 0.75, *p* < 0.001) and Main 5 (r = 0.60, *p* < 0.001), which are situated downstream. Main 5, which is upstream of Tributaries 3 and 4, shows weak correlation with these tributaries (r = 0.08, *p* = 0.0141 and r = 0.25, *p* < 0.001, respectively). In contrast, Main 6, which is downstream of these two tributaries, has higher correlation coefficients compared to Main 5 (r = 0.53, *p* < 0.001 and r = 0.34, *p* < 0.001, respectively). These results show that upstream main river sites exhibited weak or no correlation with the respective tributaries, whereas downstream sites showed strong correlations. These findings demonstrate that tributaries significantly shape the microbial community composition of the main river immediately downstream of their confluence.

## 4. Discussion

River microbial communities are an essential component of the river ecosystem, being involved in all stages of the nutrient cycle. Their study has increased exponentially with the advance of sequencing techniques. In this study, we characterized microbial communities in river sediments along the Mureș River in Romania and some of its major tributaries to evaluate the influence of tributary inflow on the bacterial community structure of the main channel. Overall, community composition and alpha diversity were comparable across sites, indicating a broadly similar level of within-site richness and evenness in both the main river and its tributaries. However, beta diversity analyses revealed clear compositional differentiation: ordination approaches (PCoA and dbRDA) consistently separated samples from the main river and tributaries, suggesting distinct community assemblages despite similar overall diversity levels. Linear regression models further supported the role of tributary inputs as significant environmental drivers shaping bacterial beta diversity. In addition, correlation analyses demonstrated a spatial pattern in which geographically proximate sites shared more similar communities, whereas the confluence with a tributary disrupted this pattern, leading to a shift in community composition that more closely resembled downstream sites. Together, these findings highlight the structuring effect of tributary inflow on sediment microbial communities in the main river continuum.

The microbial community structure observed in the river sediment samples falls within the range typically reported for freshwater benthic environments. The dominance of well-recognized bacterial phyla such as Proteobacteria, Firmicutes, Bacteroidetes, and Actinobacteria, along with the presence of archaeal taxa commonly associated with nitrogen and carbon cycling, reflects a composition consistent with previously characterized riverine sediments [15,43,44]. Overall, the taxonomic distribution and relative abundances align with patterns described in earlier studies of lotic sediment microbiomes, suggesting that the microbial assemblage in the present study represents a typical, functionally balanced freshwater sediment community rather than a disturbed or anomalous state [45,46,47].

Comparing the bacterial communities from the main river with the ones from tributaries at the phylum and class levels showed some significant differences. Most notably, at the phylum level, there was a significant difference in Bacteroidetes, with a higher abundance of this phylum in the tributaries compared to the main river. This trend was similar to observations made by Liu et al., who attributed this difference to the increased levels of dissolved oxygen observed in the main river compared to the tributaries [13]. Another observation of note was the significant increase in Cyanobacteria in the tributaries, which could be explained by the reduced turbidity of these waters compared to the ones from the main river [48]. Cyanobacteria are oxygenic photoautotrophs that require light to perform photosynthesis; therefore, reduced turbidity enhances light penetration and creates more favorable conditions for their growth. It is surprising to find that both Verrucomicrobia and Euryarchaeota increase in the same conditions, namely the main river, as they usually have different preferences. However, their increased abundance in the main river could be explained by a higher level of organic matter deposition and slower flow of the river [49,50]. At the class level, the only significant difference between main and tributaries was an increase in abundance of Actinobacteria in tributaries, potentially due to the great diversity of this bacterial class, including taxa, which can adapt to multiple environmental conditions [43].

Alpha diversity metrics indicated comparable levels of within-sample richness and evenness across all sites, with no significant differences detected between sediments collected from the main river stem and those from tributaries. This finding is not unexpected, as the sites are connected within the same fluvial network and are likely influenced by shared regional species pools, hydrological exchange, and broadly similar physicochemical conditions that can support comparable levels of microbial diversity. In contrast, beta diversity analyses revealed clear compositional differentiation between habitats. PCoA showed that samples from the main river stem clustered together, whereas tributary sediments grouped separately, highlighting differences in community structure despite similar alpha diversity metrics. This pattern aligns with findings reported by Liu et al. in the Yangtze River, where bacterial richness and evenness remained relatively stable along the fluvial continuum, yet community composition varied across stream orders [13].

Beta diversity captures variation in taxonomic composition driven by environmental gradients and spatial processes. As the stream order increases, changes in hydrology, nutrient inputs, and habitat heterogeneity can enhance the structural complexity of microbial assemblages, contributing to greater compositional turnover [6,51]. Moreover, beta diversity has been shown to be more sensitive to environmental perturbations than alpha diversity, making it a more responsive indicator of environmental influence on microbial community dynamics [52]. In this study, we evaluated the influence of selected nutrients (nitrates, ammonium nitrogen, and orthophosphates) on microbial community beta diversity and found no statistically significant effects. These results suggest that beta diversity is not driven by any single factor in isolation, but rather by the combined and potentially interacting influence of multiple environmental variables.

River sediment microbial communities are generally more stable than planktonic assemblages in the overlying water column [15,53]. This relative stability likely reflects the physically structured and chemically buffered nature of sediments, which provide redox stratification, surface attachment sites, and diverse microhabitats that promote biofilm formation and reduce dispersal-driven variability. As a result, sediment communities tend to integrate environmental conditions over longer temporal scales and exhibit lower short-term fluctuation than waterborne microbiota.

Consistent with this framework, previous work in the Mureș River demonstrated pronounced compositional stability in sediment-associated communities [23,24]. Although anthropogenic inputs, particularly effluent from the local wastewater treatment plant (WWTP), measurably influenced the river microbiome, these effects were spatially restricted to the immediate discharge zone and did not persist downstream [24]. A plausible explanation for this localized impact is that many effluent-derived microorganisms originate from human and animal gut microbiota or activated sludge communities within the WWTP and are therefore poorly adapted to riverine sediment conditions [54,55]. Lacking ecological fitness in this environment, these taxa are likely outcompeted by established resident populations, allowing the sediment community to retain its characteristic structure. In contrast, microorganisms delivered by tributaries represent river-adapted sediment taxa capable of persisting and competing within the main stem environment. Correlation analyses from the present study support this interpretation: communities downstream of confluences differed from upstream assemblages and showed greater similarity to tributary communities.

The capacity of tributaries to restructure main stem microbial assemblages is increasingly recognized as a key mechanism shaping fluvial microbiomes. O’Brien et al. (2023) demonstrated that inflows from unregulated tributaries significantly altered the bacterial community structure of a regulated river main stem, increasing diversity and shifting dominant taxa through processes consistent with microbial community coalescence and concurrent changes in physicochemical conditions [18]. Notably, communities downstream of confluences became more similar to tributary assemblages, indicating either direct taxonomic import or restructuring of resident populations [18]. This pattern closely mirrors our observations, where downstream sediment communities exhibited greater similarity to tributary communities than to upstream main stem assemblages.

Comparable trends have been reported across diverse river systems. Liu et al., investigating the Yangtze River source region, found marked differences in bacterial community composition between tributaries and main stem sites, with the stream order and tributary inputs shaping assembly patterns along the fluvial continuum [13]. Although alpha diversity remained relatively stable, beta diversity analyses revealed pronounced spatial differentiation associated with tributary influence, reinforcing the view that compositional turnover—rather than changes in richness or evenness—captures the ecological signal of confluence dynamics. Similarly, Geng et al., studying the Ili River network, reported that tributary confluences modified mainstream bacterial communities through interactions between dispersal processes and local environmental filtering, highlighting the importance of tributary origin and confluence-specific conditions in determining downstream structure [56]. Further support for this framework comes from Pan et al., who documented clear coalescence patterns between tributary and mainstream bacterial communities under varying hydrological regimes [57]. Their results demonstrate that both planktonic and sediment-associated assemblages from tributaries can contribute substantially to mainstream microbiota, depending on flow conditions and environmental context.

In agreement with previous studies, our findings suggest that tributary inputs represent ecologically compatible and competitive sources of microbial taxa capable of integrating into and reshaping main stem sediment communities. In contrast to localized and transient impacts from non-resident sources such as wastewater effluents, tributary-derived microorganisms are adapted to riverine conditions and can drive persistent compositional shifts downstream of confluences. This reinforces the role of tributaries as critical sources of microbial dispersal and community assembly within dendritic river networks.

This study has several limitations. First, only a small subset of tributaries was analyzed, which may limit the representativeness of the findings across the broader watershed. Second, sampling was conducted during a single season, precluding the assessment of potential seasonal variability in microbial community composition. Additionally, whereas some previous studies have examined both sediment and water-column microbial communities, the present study focused exclusively on sediment-associated microorganisms, based on the assumption that benthic communities exhibit greater temporal stability. Future research should adopt a more comprehensive approach by including both benthic and pelagic microbial communities, expanding the number of sampled tributaries across different stream orders—including lower-order streams—and incorporating multi-seasonal sampling to account for temporal dynamics. Such an approach would provide a more holistic understanding of spatial and seasonal variability in microbial community structure. Moreover, the analysis should also focus on the microbial function.

## 5. Conclusions

This study demonstrates that sediment microbial communities along the Mureș River and its tributaries exhibit comparable levels of alpha diversity and a taxonomic composition typical of freshwater benthic environments, with distinct assemblages between the main stem and tributaries consistently revealed by distance-based ordination and beta diversity analyses. On the other hand, tributary inflows exert a clear structuring effect on community composition, with downstream shifts in community structure affected by the influence of tributary-derived taxa. Thus, the community stability along the main river course and the restructuring effect of tributary inflows represent the two main processes that govern the spatial dynamics of sediment microbial communities in the river network. Unlike transient anthropogenic inputs, which appear spatially restricted and ecologically limited, tributary contributions represent compatible and competitive microbial sources capable of integrating into the main river sediment microbiome. These findings underscore the importance of tributaries as key drivers of microbial dispersal, community coalescence, and compositional turnover within fluvial networks, highlighting their central role in shaping sediment-associated microbial dynamics along the river continuum.

## Figures and Tables

**Table 1:** Alpha diversity indexes for the bacterial communities from each sampling site.

Sites	Species No.	ACE	Chao1	Shannon	Gini–Simpson	Pielou
Main 1	437	461.1093	467.1539	4.871657	0.983881	0.801268
Main 2	486	514.3232	508.3594	4.711286	0.975178	0.761579
Main 3	388	421.7674	419.3182	4.507695	0.975707	0.756197
Tributary 1	381	420.5398	427.8837	3.535411	0.892502	0.594907
Main 4	348	378.9167	376.2	4.145589	0.955936	0.708381
Main 5	250	286.4108	285.8378	2.803965	0.876217	0.50783
Tributary 2	442	461.3878	460.3191	4.528365	0.971141	0.743414
Tributary 3	518	542.4336	543.0213	5.287107	0.991096	0.84594
Tributary 4	418	436.2827	438.6765	5.00658	0.986947	0.829525
Main 6	401	425.2088	428.7742	4.800979	0.982488	0.800969
Main 7	479	506.2642	501.6852	5.027248	0.983328	0.814565
Main 8	315	338.3771	332.6429	3.301878	0.82421	0.573983
Main 9	490	562.1718	560.9492	4.656634	0.977988	0.751748

## Data Availability

The original data presented in the study are openly available in the NCBI GenBank/NCBI Sequence Read Archive (SRI) under the accession number PRJNA1439182 (http://www.ncbi.nlm.nih.gov/bioproject/1439182, accessed on 30 March 2026).

## Abbreviations

The following abbreviations are used in this manuscript:

ASV	Amplicon sequence variant
dbRDA	Distance-based redundancy analysis
PCoA	Principal coordinate analysis
